# The evaluation of ^137^Cs radioactivities in soils taken from the Babia Góra National Park

**DOI:** 10.1007/s10967-013-2809-z

**Published:** 2013-11-05

**Authors:** M. Stobinski, K. Szarlowicz, W. Reczynski, B. Kubica

**Affiliations:** 1 Faculty of Energy and Fuels, AGH University of Science and Technology, al. A. Mickiewicza 30, 30-059 Kraków, Poland; 2 Faculty of Material Science and Ceramics, AGH University of Science and Technology, A. Mickiewicza 30, 30-059 Kraków, Poland

**Keywords:** ^137^Cs, Babia Góra National Park, Soil cores, Cluster analysis, Principal component analysis

## Abstract

The aim of this work was to determine ^137^Cs and ^40^K radioactivities in soil samples taken from the Babia Góra National Park (BPN) in south Poland. The cluster analysis (CA) and principal component analysis (PCA) were used to discuss the obtained data. 10 cm thick soil cores were collected from the BPN area. Each sample was divided into three sub-samples. The samples were dried, homogenized and packed in polyethylene containers. The radioactivities of ^137^Cs and ^40^K were measured by means of gamma spectrometry. It was found that ^137^Cs radioactivity in the whole 10 cm soil cores was in the range from 1,916 to 28,551 Bq m^−2^. The radioactivity of ^40^K varied from 1,642 to 25,654 Bq m^−2^. Using CA it was possible to diverse the soils taking into account soil types. By use of the PCA method, it was chosen three factors which are appropriate to characterize researched parameters.

## Introduction

Mountainous ecosystems are highly sensitive to any disturbances in natural balance, what makes them especially interesting for observations of chemical and radiochemical contamination. The area of the Babia Góra National Park (BPN), being located along the main ridge of the Flysch Carpathian Mountains, is just the proper place in this respect. The terrain belongs to the young folded mountains, built mainly of flysch and some other lithology. The area is characterized by highly variable and unique natural resources. The highest summit of the Babia Góra Mountain’s massif (west part of the Beskid Żywiecki chain of mountains) is Diablak reaching 1,725 m asl. To protect this unique area the BPN and the UNESCO World’s Biosphere Reserve were established. The area of the BPN, like other regions of Poland, was exposed to substantial contamination with radionuclides due to the Chernobyl accident in 1986 and nuclear weapon tests since the fifties of the twentieth century [1–6]. Researches on radionuclides distribution in the environment are important not only because these are perfect markers of the environment pollution but also because radioactive isotopes are not immobilized in soil but are constantly interchanged between inorganic matter and living organisms [7].

The aim of the work was to establish the spatial distribution of γ-radionuclides i.e. anthropogenic ^137^Cs (*T*_1/2_ = 30.07 years) and natural ^40^K (*T*_1/2_ ≈ 10^9^ years) in the soil samples collected in the described above area.

## Materials and methods

10 cm thick soil core samples were collected with the use of a cylindrical corer (10 cm in diameter). 13 sampling points were selected in the studied area, most of them localized along the mountains’ main ridge. Each of the sampling points was described by its geographical coordinates and height above the sea level. In case of few points it was impossible to determine their precise coordinates (the lack of GPS signal), so the closest available coordinates were interpolated. In the laboratory, the cores were divided into three sub-samples each to enable radioisotopes determination in various depths. The core sub-samples were marked as follows—“a” (0–3 cm), “b” (4–6 cm) and “c” (7–10 cm). The samples were then dried at 105 °C and the total weight was determined. After removal of organic macro-particles and small stones, the samples were sieved mechanically (2 mm mesh).

After preparation, the samples were analysed by means of gamma ray spectrometry with the use of HPGe (high purity germanium) coaxial detectors of relative efficiency 7 and 21 %. Prior to the measurements, the soil density was determined for further use in calibration corrections calculations [8]. Each measurement lasted 72 h. The reference materials IAEA-154, IAEA-375, and IAEA-447 of the International Atomic Energy Agency (Vienna, Austria) were used in the measurements. In the present work ^137^Cs radioactivity in the “a” soil layer was given in Bq kg^−1^ units. For the whole core samples, ^137^Cs and ^40^K activities were expressed in Bq m^−2^ units. All given caesium radioactivity values were recalculated for the day 01.09.2010. After gamma spectrometric measurements organic matter content in the soil samples was determined by means of incineration at 600 °C.

To extract relevant information out of the obtained data, the data matrix consisting of all analysed features of all samples was analysed statistically. Chemometric tools i.e. cluster analysis (CA) and principal components analysis were used (using Statistica 10 software).

## Results and discussion

The sampling points description is given in Table 1. The results of radioisotopes activity and soil density are presented in Table 2. The most important values are written in bold.

**Table 1:** The Babia Góra National Park sampling points description

Point no.	Geographical coordinates	Height (m asl)	Place description
1	N 49st 34,797	1,515	Red hiking trail, after Sokolica, right from this red route
	E 19st 33,414		
2	N 49st 34,616	1,577	Near Zimny Staw Lake, at the top of Babia Góra
	E 19st 32,772		
3	N 49st 34,341	1,707	By green hiking trail from Babia Góra to Lipnica
	E 19st 31,824		
4	N 49st 34,426	1,701	The yellow hiking trail from Babia Góra, on the right sight of this route
	E 19st 31,769		
5	N 49st 34,386	1,695	The route from Diablak (red hiking trail)
	E 19st 31,689		
6	No data	1,600	The route from Diablak below Cross (red hiking trail)
7	No data	1,530	Krzyżówka szlaków na Markowe Szczawiny i Małą Babią.
8	No data	950	Mokry Stawek Lake, from the site of Krowiarki Pass
9	No data	951	Brona Pass
10	N 49st 34,528	1,463	By yellow hiking trail, dwarf pine zone
	E 19st 31,453		
11	N 49st 34,667	1,350	Yellow hiking trail, at the end of higher montane zone
	E 19st 31,301		
12	N 49st 34,953	1,257	At the intersection of Szumiąca woda Valley with the route to Markowe Szczawiny
	E 19st 31,467		
13	N 49st 34,293	836	Hala Śmietanowa, above little bridge on Syhlec stream
	E 19st 35,609

**Table 2:** ^137^Cs and ^40^K radioactivities, density and organic matter content in the soil samples collected in the Babia Góra National Park area

Point no.	Height (m asl)	Soil density (g cm^−3^)	Organic matter content (%)	Activity ^137^Cs (Bq kg^−1^) (layer “a”)	Activity ^137^Cs (Bq m^−2^) (whole core)	Activity ^40^K (Bq m^−2^) (whole core)
1	1,515	0.70	17	416 ± 11	8,837 ± 241	6,900 ± 619
2	1,577	0.17	90	405 ± 16	3,757 ± 211	4,127 ± 374
3	1,707	0.55	20	304.1 ± 9.2	6,591 ± 224	7,914 ± 715
4	1,701	0.65	15	**831** ± 21	**28,551** ± 720	16,477 ± 1,497
5	1,695	0.70	14	246.2 ± 8.4	8,166 ± 275	10,848 ± 977
6	1,600	0.24	73	697 ± 21	7,231 ± 219	2,134 ± 194
7	1,530	0.22	81	214 ± 10	3,276 ± 177	7,869 ± 713
8	950	0.99	9	36.8 ± 2.1	1,783 ± 119	**25,654** ± 2,288
9	951	0.,70	37	65.8 ± 6.4	2,119 ± 152	12,907 ± 1,164
10	1,463	0.50	21	137.3 ± 6.1	1,916 ± 83	8,368 ± 775
11	1,350	0.57	19	309 ± 10	5,472 ± 230	**25,200** ± 2,255
12	1,257	0.17	82	326 ± 15	2,030 ± 95	1,642 ± 151
13	836	0.40	23	408 ± 13	16,152 ± 484	17,480 ± 1,573

Soil in the BPN originates from the Carpathian Flysch waste. As in the area vegetation levels are well distinguished, for each sampling point the soil type can be attributed. The resulting soil type classification is given in Table 3 [9].

**Table 3:** Prevailing soil types of the considered sampling points

Vegetation level	Height (m asl)	Prevailing soil type	Sampling points
Alpine zone	1,650–1,725	Lithic Leptosol,	3, 4, 5
		Regosol Skeletic,	
		Podzolic Ranker (Leptosol)	
Dwarf pine zone	1,390–1,650	Haplic Podzol,	1, 2, 6, 7, 10
		Tangel–Ranker	
		(Umbric Leptosol)	
Higher montane zone	1,150–1,390	Dystric Cambisol	12, 11
		Partially Haplic Podzol	
Lower montane zone	700–1,150	Eutric and Dystrict Cambisol	13, 8, 9

**Fig. 1:**
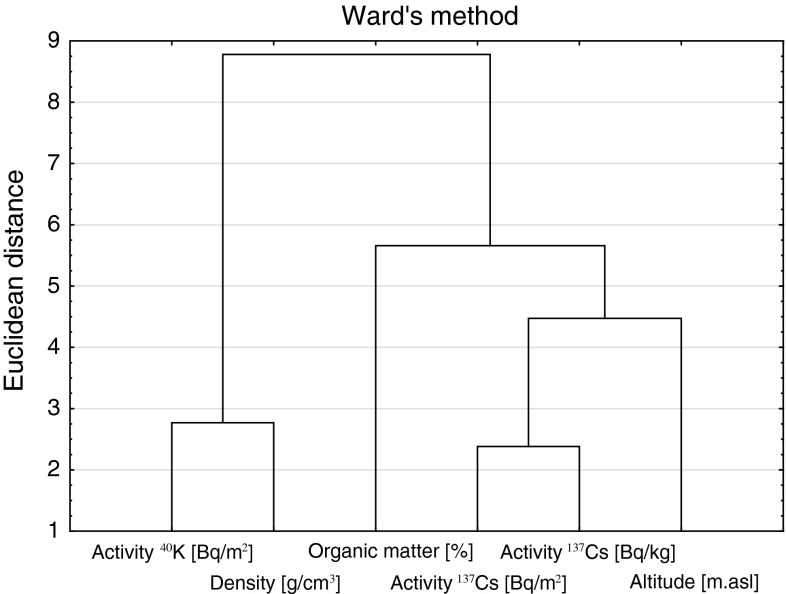
Cluster analysis (variables). Activity of ^137^Cs [Bq·m^−2^] represents activity in the whole profile; activity of ^137^Cs [Bq·kg] represents activity in “a” layer

**Fig. 2:**
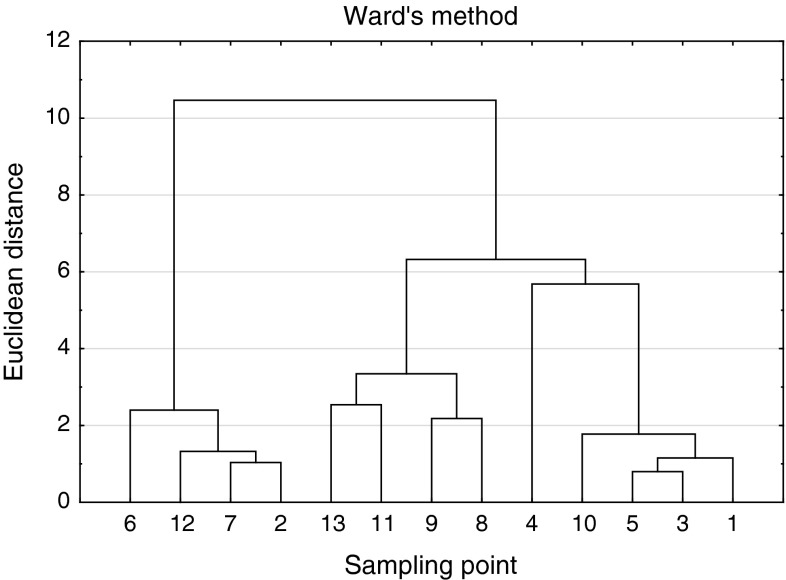
Cluster analysis (samples)

Chemometric tools are widely used in the environmental data analysis especially when high variability and uncertainty of the data may be expected [10]. For the obtained set of data the CA according to Ward and principal components analysis (PCA) after initial normalization/auto-scaling, were used. The outcome of the analyse is presented in Figs. 1 and 2.

In Fig. 1 the dendrogram of the variables similarity is analysed. Two main clusters are distinguished—the first one represents soil density and ^40^K radionuclide activity (^40^K activity is in constant relation to the total potassium, about 31 Bq ^40^K per 1 g of potassium [11]).

These two variables belong to the same cluster what suggests that potassium is incorporated mainly in mineral components of the soil. The higher is mineral content of soil, the higher is soil density (Table 2). In the second cluster, radioactivities of artificial ^137^Cs in the “a” soil layer and in the whole core as well as the sampling point height asl and organic matter content, are grouped.

Similar course of changes of ^137^Cs activity and soil organic matter content can be attributed to the sorptive properties of this soil fraction. As ^137^Cs presence in the soil results only from the distant transport of the contaminant, its retention in soil is a direct result of its sorption. The other important factor is the amount of precipitation in the area (rain, snow). The higher is the location of the sampling point in the mountains, the higher is the amount of precipitation. Thus, the variable—altitude—belongs to the same cluster.

In Fig. 2 the similarity of sampling points is presented. The clusters group points not necessarily close to each other. But the chosen criteria i.e. analysed variables, enabled proper and logical division of the sampling points into groups (clusters) for which the soil type is a common feature (Table 3). However, it should be noted that in mountains, soil types are not sharply defined. As a result the revealed clusters do not classify soil types directly, but the obtained classification is relevant for a given soil complex structure (Fig. 2; Table 3).

In further statistical analysis of the obtained results, the principal components analysis was performed. Principal components are the linear combination of the previous variables.

**Fig. 3:**
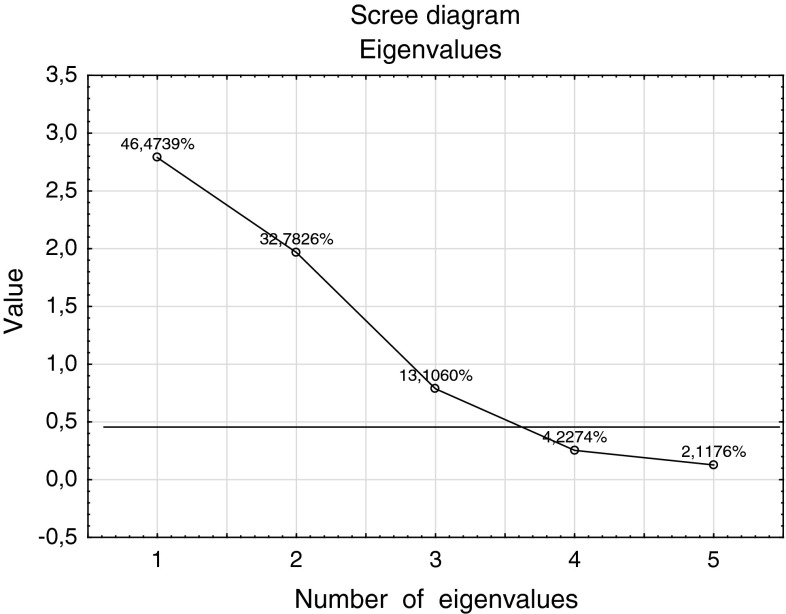
Scree diagram

Taking into consideration the Kaiser criterion in further analysis only the first and the second principal component should be used (Table 4). The scree diagram suggests however, that it would be reasonable to consider three components as they describe 92.36 % of global variation of the data (Fig. 3).

In Table 5 factor loadings (after varimax rotation) for the three principal components are given.

**Table 4:** Eigenvalues

	Principal components analysis			
	Eigenvalues	% total variance	Cumul.-eigenvalue	Cumul.-%
1	2.79	46.48	2.79	46.48
2	1.97	32.78	4.76	79.26
3	0.79	13.10	5.55	92.36
4	0.25	4.23	5.80	96.59
5	0.13	2.12	5.93	98.71
6	0.07	1.29	6.00	100.00

**Table 5:** Factor loadings (varimax)

	VF-1	VF-2	VF-3
Altitude	−0.090	0.232	**0.942**
Density	**0.950**	−0.107	−0.096
Radioactivity of ^137^Cs (“a” layer)	−0.235	**0.906**	0.271
Radioactivity ^40^K	**0.721**	0.094	−0.556
Radioactivity ^137^Cs (whole core)	0.247	**0.945**	0.025
Organic matter	**−0.956**	−0.108	0.050

Significant values are given in bold

The first component (explaining 46 % of variability) covers mainly the following variables: density, organic matter content and potassium concentration. All these parameters are connected with the physical properties and chemical composition of the soil. The second principal component, explaining 33 % of variability, considers only anthropogenic variable i.e. radioactivity of ^137^Cs. Third principal component explaining 13 % of variability refers to the height asl of the sampling point. It should be stressed, that this component supplies additional information related to some aspects explained by the first two principal components. The height above sea level has the influence on such soil properties like density and organic matter content. With increasing altitude the pace of organic matter decomposition decreases (the same tendency is noted for temperature). Simultaneously, anthropogenic ^137^Cs was introduced to the soil mainly with rainfall—in the mountains, the higher located is the place the more rain is precipitated.

**Fig. 4:**
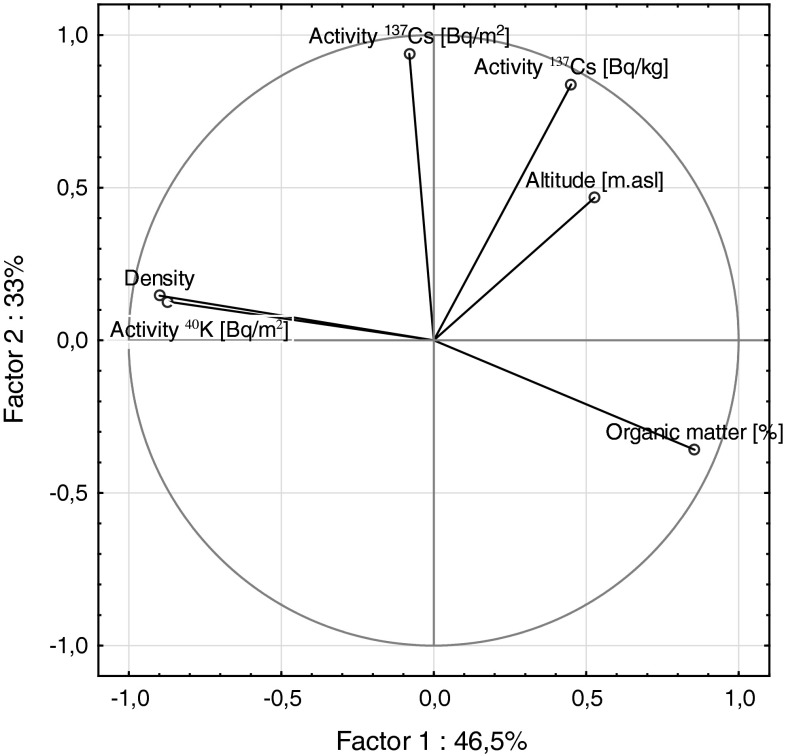
Projection of the variables onto the plane of the first two principal components

In Fig. 4 it may be noted that the variables (soil density and potassium content) are positively correlated witch each other and negatively correlated with organic matter content. On the other hand it is clear that ^137^Cs radioactivity is orthogonal to those variables (soil density and potassium content)—no correlation was found.

## Conclusions

It was established that:

- Caesium radioactivity in the soils of the BPN is changeable. In the top (“a” layer) 3 cm thick soil layer it is in the range from 36 to 831 Bq kg^−1^, while in the whole core (10 cm) it spans from 1,916 to 28,551 Bq m^−2^. CA enabled grouping of the sampling points according to the soil type. It may be assumed that the sampling points are better characterized by the ^137^Cs radioactivity expressed in surface units rather than radioactivity of the first layer only.
- The PCA reduced the number of analysed variables to three principal components, explaining 92 % of the total variance of the variables. First three principal components differentiate the variables into: natural (connected with physical and chemical soil properties) like soil density, organic matter and potassium contents, anthropogenic—^137^Cs radioactivity, and sampling points characteristic—altitude above sea level.

Presented above conclusions are concomitant with the results of research performed in the similar mountainous ecosystems. It is true especially for the neighbouring Tatra Mountains National Park [3, 5, 12]. The similar positive correlation of ^137^Cs activity with the sampling point altitude and organic matter content in the soil were found in the Tatras. Similarly the soil density was correlated with potassium content.

Soils of the mountains neighbouring to the BPN constitute currently the subject of complementary research projects.
